# A Boolean Model of the *Pseudomonas syringae* hrp Regulon Predicts a Tightly Regulated System

**DOI:** 10.1371/journal.pone.0009101

**Published:** 2010-02-15

**Authors:** Daniel MacLean, David J. Studholme

**Affiliations:** The Sainsbury Laboratory, John Innes Centre, Norwich, United Kingdom; University of Wisconsin-Milwaukee, United States of America

## Abstract

The Type III secretion system (TTSS) is a protein secretion machinery used by certain gram-negative bacterial pathogens of plants and animals to deliver effector molecules to the host and is at the core of the ability to cause disease. Extensive molecular and biochemical study has revealed the components and their interactions within this system but reductive approaches do not consider the dynamical properties of the system as a whole. In order to gain a better understanding of these dynamical behaviours and to create a basis for the refinement of the experimentally derived knowledge we created a Boolean model of the regulatory interactions within the hrp regulon of *Pseudomonas syringae* pathovar tomato strain DC3000 *Pseudomonas syringae*. We compared simulations of the model with experimental data and found them to be largely in accordance, though the hrpV node shows some differences in state changes to that expected. Our simulations also revealed interesting dynamical properties not previously predicted. The model predicts that the hrp regulon is a biologically stable two-state system, with each of the stable states being strongly attractive, a feature indicative of selection for a tightly regulated and responsive system. The model predicts that the state of the GacS/GacA node confers control, a prediction that is consistent with experimental observations that the protein has a role as master regulator. Simulated gene “knock out” experiments with the model predict that HrpL is a central information processing point within the network.

## Introduction

Many gram-negative bacterial pathogens of plants use the Type III Secretion system (TTSS) to deliver effector molecules directly into the host [1]. The TTSS is a tube like structure with ring structures embedded in the plasma membrane and a filament structure termed the *hrp* pilus [2], [3]. The TTSS is encoded by around 20 hrp (hypersensitivity response and pathogenicity) genes that are found in several operons on the chromosome or plasmids of plant-pathogenic bacteria [4]. The hrp operons are grouped according to operon structure. The *hrp* genes of *Pseudomonas syringae* pathovar tomato strain DC3000 (*P.syringae*), *Erwinia* spp. and *Pantoea stewartii* belong to group I [5]. Here we consider this group, specifically the signal transduction components [5]–[16] influencing expression of the *hrp* regulon have been discovered in *Pseudomonas syringae*.

The regulatory interactions in the *hrp* regulon are reviewed in detail elsewhere [5]. Briefly, in *P. syringae* the GacS sensory histidine kinase and GacA cognate response regulator dimer activate transcription of the alternative sigma factor gene *rpoN*, which together with the dimer HrpRS (a member of the NtrC family of two-component regulator proteins) regulates transcription of the *hrpL* gene. HrpL activates transcription of the *hrp* regulon via interactions with the hrp box, a conserved nucleic acid sequence promoter motif and RpoN. Together these interactions result in the expression of the genes encoding proteins that constitute the machinery of the TTSS.

Thanks to the concerted efforts of many groups a good number of the signal transduction and regulatory components of the hrp genes in *P. syringae* and their interactions with each other have been discovered [5] but the dynamics of the system as an integrated whole have not been considered. In this study we hoped to use modelling techniques to synthesise the biochemical and molecular information available and take the next step. The approach we intend can be understood with a simple analogy: the powerful reductionist approaches of traditional biochemistry and molecular biology are analogous to a watchmaker deconstructing a watch, listing the component parts but perhaps stopping at drawing a picture of how it goes back together. The logical next step for the watchmaker is simply to put the watch back together, wind it up and see if it goes. The availability of molecular information and modelling methods allow the biologist to take this next step. Modelling the interactions allows biologists to understand a whole new level of operation of a system of interest, to observe emergent features that are not obvious from the parts list and known interactions alone. When modelled scenarios do not match up with the results observed in real life, discrepancies may not be due to weaknesses in the model. Rather they can indicate errors in our understanding of regulatory relationships or omissions from the parts list and allow us insight that can result in the refinement of our knowledge.

In recent years mathematical models have been applied to the computational analysis of biochemical networks including metabolic pathways, signal transduction and gene regulatory networks [17]–[19]. In situations where the biochemical and kinetic parameters of a system are known in great detail modelling with continuous or stochastic approaches such as differential equations or Monte Carlo simulations [20] is informative and appropriate. Often the amount of information on biochemical parameters is very low indeed and stochastic or continuous models cannot be formulated. In the absence of detailed biochemical information, discrete deterministic, parameterless models can be constructed. Such models are being used increasingly to reproduce dynamical behaviours of molecular control networks and it is being discovered that it is the sequence of events rather than the timing that is the important factor [21]–[27], thus much can be learned from these sorts of models. One such class of discrete model is the Boolean model. Boolean representations are very common in biology and are often used implicitly for describing sets of regulatory interactions in diagrams and figures describing models so they are a natural tool for biologists to analyse and interpret. In the Boolean formalism a network is created with the entities under study as nodes and regulatory relationships as one-way (directed) links between them. Nodes can have two states; True or False. As the model is run the states of each node are updated according to the states of the upstream nodes via a set of update rules represented as a logical statement using Boolean operators AND, OR, NOT that evaluate to either True or False. The changing pattern of states that the nodes pass through during the time evolution of the model is called its dynamical trajectory. We wanted to know whether a Boolean model could be used to reliably reproduce the observed patterns of expression of the genes of the hrp regulon and then if it could be used to identify any interesting dynamical properties of the system that were not obvious from the literature.

## Results and Discussion

### A Discrete Dynamical Model of the *hrp* Regulon of *Pseudomonas syringae*

There is a paucity of kinetic and quantitative information on biochemical parameters such as protein DNA binding affinities, RNA polymerase extension rates and so on in the specific hrp regulon literature, so it is not possible to create detailed continuous or stochastic models without making gross and probably erroneous estimations about the values of these parameters. We used a discrete Boolean model framework to create a model of, and to simulate the activity of the hrp regulon.

**Figure 1:**
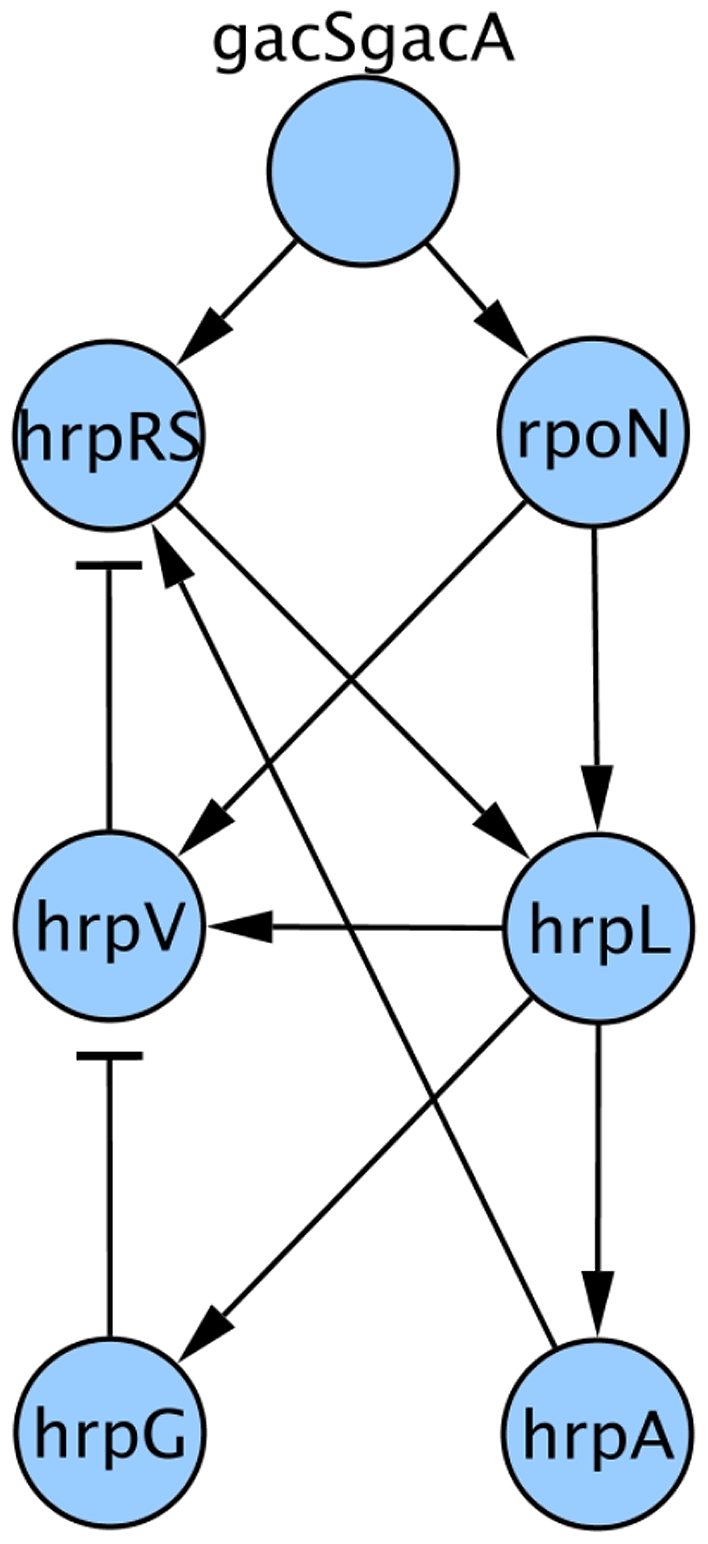
Network of the hrp regulon of *P. syringae*. Nodes (Blue circles) represent the proteins in the network and edges (black lines) represent regulatory interactions, arrow headed edges represent a positive regulatory interaction and T-headed edges represent a negative regulatory interaction.

The regulon was reproduced as a directed network by combining literature data (summarised in [5]) for *P.syringae*, into an interaction network (Figure 1). We decided that proteins are the entities under study within the network and they are represented as nodes. Genes and mRNA are implicitly contained in these nodes. Regulatory interactions are represented as directed edges starting in the source, regulator node and ending in the target regulated node; regulatory interactions are classified as either activation or inhibition and are represented by arrows or blunt ends to edges respectively. In the spirit of making our model as simple as possible, but not any simpler, the choice of nodes and interactions was made so as to minimise the complexity without losing essential information. This meant removing nodes that were redundant, i.e. functionally equivalent to another such as the genes of the hrp regulon that according to our knowledge have no effect on other genes within it, or merging some proteins into single nodes because their dimerisation is required for action. The pairs of proteins GacS and GacA and also HrpR and HrpS are understood to have regulatory roles only when they have heterodimerised [13], so we condensed these pairs into just two nodes representing the heterodimers GacSGacA and HrpRS, which has no effect on the dynamics of the network and removes needless complexity. Other factors that may be presumed to be constant between the different genes can be ignored in the model. Some factors that are not truly constant between genes *in vivo*, like transcription and degradation rates but in which the differences are due to time dependent factors end up being equivalent because of the Boolean framework. The complexities of RNA Polymerase holoenzyme formation and alternative sigma factor RpoN regulation while itself complex, boils down to whether or not the RpoN protein is present and need not be considered in more detail. Our network contains 7 nodes; GacSGacA, RpoN, HrpRS, HrpV, HrpA, HrpG and HrpL representing 9 proteins.

We used information from the literature to compose a set of state-change rules that were formulated according to Boolean rules (Table 1). Each node in the model has a binary state, either True (1, or on) or False (0, or off), reflecting the eventual expression of the gene. A nodes state depends on the states of the nodes that lead to it; that is the state of a protein is a function of the state of the proteins that have regulatory action up on it. We used the AND operator when literature reports state that multiple proteins are required for activation, the OR operator when only one of a number is sufficient to activate and the AND NOT operator when a protein inhibits another. The protein GacGacA has no state-change rule because it is the most upstream protein and none of the other proteins in the model act upon it and no other proteins are known to regulate it in *P. syringae*, though HrpXY is known to regulate GacSGacA in *Erwinia* and *Pantoea stewartii*.

**Table 1:** Network nodes in the model and corresponding Boolean update rules.

Node	Update rule
GacSGacA	-
RpoN	GacSGacA
HrpRS	(GacSGacA) and (not HrpV) or (HrpA)
HrpV	(HrpL and RpoN) and not HrpG
HrpA	HrpL
HrpG	HrpL
HrpL	RpoN AND HrpRS

### Dynamical Model of the *Pseudomonas syringae* hrp Regulon Recreates Patterns of Expression Observed *In Vivo*

**Figure 2:**
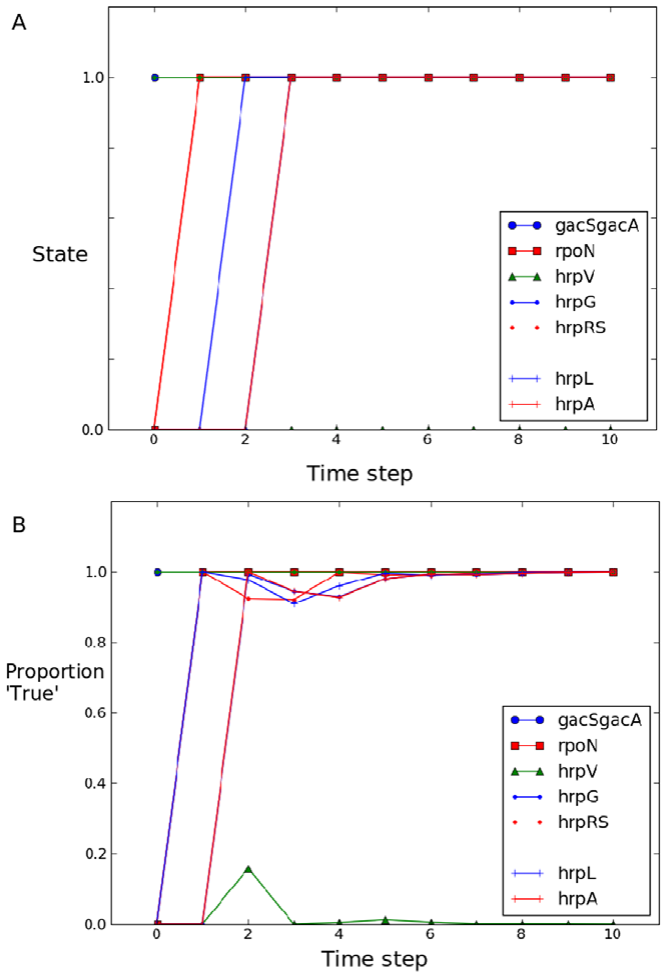
Time evolution of proteins in the model. We ran the model in synchronous mode (A) and examined the state of each protein at each time step for 10 steps. 1 = True, 0 = False. We also ran the model in asynchronous mode (B) for 10,000 repeat runs and calculated the proportion of runs in which each protein was in the True state.

After constructing the model we considered the time evolution (the change of state over time) of the proteins. We ran the model by setting initial conditions for all proteins to False, with the exception of GacSGacA which was set to True. We ran the model for 10 steps in synchronous mode [28], which assumes that all regulatory processes have the same duration, and that there is time for only one update of each protein's state within each time step of the model. The time evolution of the proteins in the synchronous runs of the model can be seen in Figure 2A. The proteins are activated (obtain a state of True, or 1) in a specific sequence, RpoN and HrpRS are activated by GacSGacA immediately and in turn activate HrpL at the second step. HrpL is then able to activate the other proteins of the regulon and the system reaches a steady state with all proteins, except HrpV activated by the third step. This pattern matches very well the observed pattern of expression and expression patterns of the genes of the hrp regulon and would be quite unlikely to occur from the model by chance, given the potential state space. The False state of HrpV reflects the structure of the model rather than the biological situation, HrpV is regulated negatively as a protein by HrpG in *P. syringae* and this is reflected in our model, but accumulation of *hrpV* mRNA can occur independently. The model doesn't clearly represent the situation where the turn over or functional status is changed.

### Wiring Is More Important Than Timing in the Model of the hrp Regulon

One interesting question to ask of the model is whether the behaviours it displays are dependent on the timing of the events within it or whether only the sequence of events is important. Systems that are time independent are more robust to stochastic perturbations. So to determine whether the timing of regulatory processes was critical to the overall time evolution of node state we ran the model in an asynchronous update mode. In this the mode the time scale of the regulatory processes are randomly chosen [28]. This is achieved by updating node states in a random order rather than in a predetermined one, and we record a time step as the longest interval for a node to respond to changes in its regulators. Asynchronous updates introduce a stochastic dimension to the evolution of the system [29], [30] and can vary the steady states reachable from the initial states of the system. We ran the model from the same starting point as the synchronous model described above for ten steps and with 10,000 iterations. For each protein at each time step we calculated the fraction of runs that had a value of True at that step (Figure 2B). In the asynchronous update run HrpRS, RpoN and HrpL activated immediately in 100% of runs and stayed activated for 92% or more of runs thereafter. The other proteins activate in more than 92% of runs one step later. Again, HrpV remains in an off state for the entire run, being activated in only 16% of runs at step 2. The similarity in the evolution patterns of the synchronous and asynchronous runs is striking; both reach an essentially steady state in which the hrp regulon is expressed after 2 or 3 steps. This indicates that the timing of regulation is less important than the ‘wiring’ of the system in specifying its dynamical behaviour so we were able to use the less computationally expensive synchronous updates of the model for all subsequent runs which is a useful technical aspect but it implies a much more subtle point. Systems that are not dependent on timing are much more robust to the sorts of stochastic variation that one would expect in biochemical systems, the advantage of this is straightforward, it ensures that the expression of the TTSS will occur. Robustness of this sort also protects the functioning of the system against evolutionary change in the sequences of the proteins of the TTSS. Changes in promoter, gene and protein sequences are all able to affect the rate and steady state level of gene expression, which could really disturb functioning in a time-dependent system. In a robustly wired system sequences are more free to evolve with less chance of disrupting system function. Such freedom could be essential in a system that specifies a crucial part of the pathogen infection apparatus as this, allowing it to evolve in response to changes in the host if needed.

### The Model Predicts That the hrp Regulon Cannot Be Accidentally Activated by Ectopic Expression of Genes Within It and GacSGacA Is the Only Determinant

**Figure 3:**
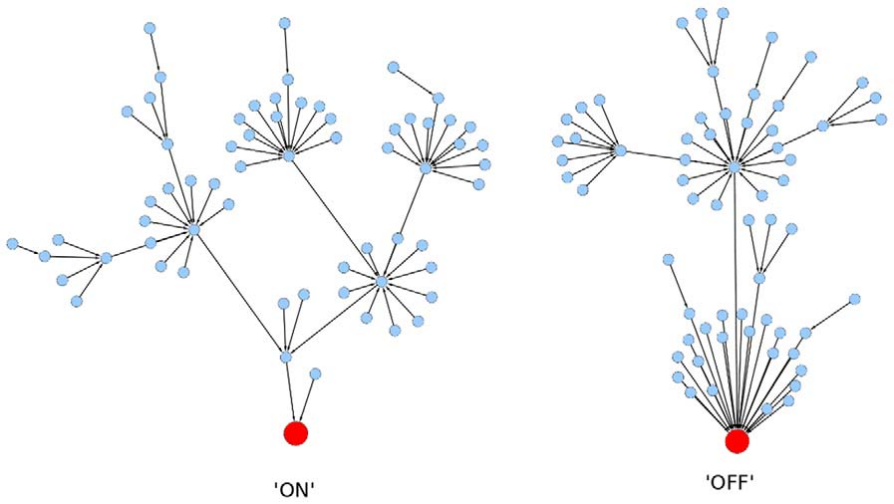
Attractor trees of the model for the 128 different start states. We ran the model in synchronous mode starting from each of the 128 possible combinations of states. Each circle represents a possible state of the model and the edge indicates the state to which the model evolves on the next iteration. The tree with the terminal node labelled ‘ON’ has an attractor with the same state as the steady state of runs with the model i.e GacSGacA  =  True; RpoN  =  True; HrpV  =  False; HrpG  =  True; HrpRS  =  True; HrpL  =  True; HrpA  =  True; The tree with terminal node labelled ‘OFF’ has an attractor in which all states are false.

So that we might understand the paths through which the system could possibly run, we ran the model in synchronous mode for 10 steps starting from each of the 2 (128) possible states of the model and mapped the dynamical trajectories from each start state to the final state via every state the model occupied on the way. Such an analysis provides a map of the way the system could possibly behave under different combinations of ectopic expression of its genes. The results of this analysis can be seen in Figure 3. In Figure 3 each of the dots represents a model state and the arrows lead from a state to the subsequent state. Remarkably, the model converges on two discrete end-points or attractors. The two discrete trees that lead to an attractor each contain 64 states, 50% of the total. One of the trees leads to an attractor identical to the steady state describe above, with all the proteins (with the exception of HrpV) showing a state of True. The second tree leads to an attractor with all proteins in the False state. A similar analysis averaging 10,000 runs in an asynchronous mode showed the same pattern. The presence of just two attractors indicates that the system is a strongly regulated switch, optimised to allow only expression of the components of the hrp regulon all together or not at all, predicting that a non-constitutive mutation in expression of any combination of genes cannot cause ectopic expression of the hrp regulon. To ascertain whether or not a specific factor or factors in the dynamical model could be determinants of which attractor a state leads to, we calculated for each protein the number of times it was true or false for each step of the evolution of the model. We did this starting from each possible start step in each of the two attractor trees described above. During the initial states of the runs for both attractor trees, each protein, except GacSGacA could be in either state, in fact at the start of runs all proteins except GacSGacA were equally in either state. The state of GacSGacA throughout the runs corresponds to the final state in each of the attractor trees, when GacSGacA is True the model is attracted to an ‘on’ steady state regardless of other perturbations, and when GacSGacA is False the model attracts to an ‘off’ state. This indicates that GacSGacA is the sole determinant of the expression of the genes of the hrp regulon and that ectopic expression of other components cannot initiate or sustain the expression of the regulon. Such an observation is intellectually satisfying firstly because it reflects the situation observed *in vivo* but secondly, and more importantly, it reflects a system that is not capable of being accidentally ‘hot-wired’ by changes in its components expression patterns. Therefore expensive accidental deployment of the TTSS machinery and effectors is not likely to occur because of short-circuiting of the system itself. The GacSGacA dependency is both a strength and a weakness. Although the pathogen is able to deploy its TTSS according to specific inputs and is not likely to accidentally misfire, hosts that are able to disrupt the activation of GacSGacA are able to prevent the activation of the TTSS.

### Simulated Knock-Outs Predict Essential Proteins Within the *hrp* Regulon Model

**Figure 4:**
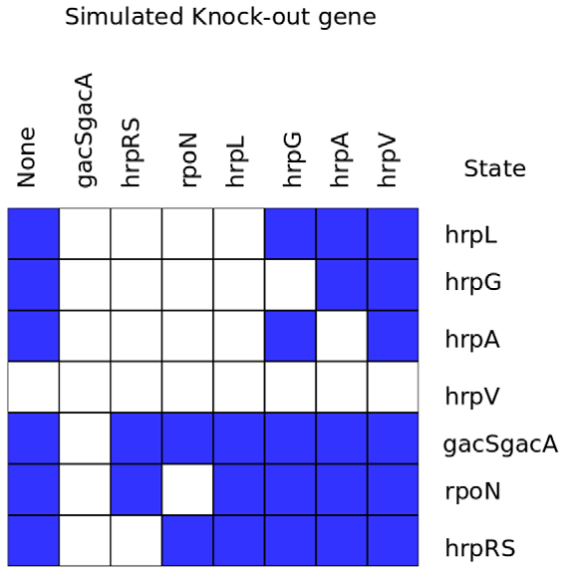
States of the model at step 10 in runs with simulated knock-outs of individual genes. We ran the model in synchronous mode for 10 steps from the initial state and simulated a knock-out of a single gene, recording the model's state at step 10. Each column represents the results from a single run with a single knocked out gene, indicated above the column, each row represents a gene. Blue colour indicates that the model showed the gene was in the ‘True’ state at step 10, no colour indicates the model showed ‘False’ for the protein at step 10.

To find essential nodes in the model it is possible so to determine whether any proteins in the model could be essential to the normal steady state that we have already described above and in Figure 1 we performed synthetic knock-outs, running the model synchronously with a single protein's state set to False throughout the run. Figure 4 shows the results of this analysis. In Figure 4 each of the columns represents the tenth state for a run with the protein at the head of the column knocked-out. A blue cell indicates that the protein on the row was in the True state, a white cell indicates that the protein was in the False state. Absence of GacSGacA leaves the model in the ‘off’ attractor state. The analysis reveals proteins HrpRS, RpoN and HrpL are all required for the expression of the other hrp proteins in the model, but are not dependent on them for their own expression. As HrpL is directly downstream of both HrpRS and RpoN and only activates state when both inputs are True it can be considered that HrpL functions as an integrator of these two inputs, requiring that both are received for the activation of the rest of the regulon. It could be argued that the pathogen would easily be able to circumvent these switches by over expressing a single component, such as HrpL, but this would result in constitutively expressing the TTSS and would likely to be disadvantageous.

### Conclusions

The identification of missing parts and connections is only one possible new source of information that a model can give us, the emergent and dynamical behaviours of the system cannot be appreciated from the network diagrams common in the biological literature. Modelling studies allow us to identify behaviours not predictable from the network diagram. The dynamical properties of the hrp regulon have not been studied before and the Boolean model we have created is able to reproduce the pattern of changes observed *in vivo*. The Boolean model predicted that the system is constructed such that wiring is more important than timing and that the ectopic expression of the components cannot accidentally activate the regulon. These time-independent dynamics also allow for evolutionary change within the components of the system themselves without adversely affecting the functioning of the system as a whole. Such an arrangement would be useful for biological systems that rely on interactions between molecules whose primary sequence is liable to alter by chance mutation which could alter stochastic properties and systems would need to evolve protection against this to remain robust. Evolving a network where the connections specify the behaviour is one way to retain robustness and evolvability. The Boolean model confirms that the system is dependent on the specific GacSGacA ‘switch’ that had been shown experimentally but it was also able to show that no other protein or combination of proteins within the hrp system is able to take over the role of GacSGacA and specify an ‘on’ state of the system.

## Materials and Methods

The network of signalling components of the hrp regulon was constructed by compiling information from an extensive literature study from the following primary reports([5]–[16]. The update rules described in Table 1 were compiled based on the interactions described in the reports and summarised in [5]. Once this was done we were able to formulate the rules and create the model in a Boolean framework ready for simulation. Model construction and simulation was carried out in the BooleanNet 1.2.4 system [28], a Python scripting language software library that allows the definition of a model by listing the entity and update rules described above. The BooleanNet system is capable of interacting with standard Python plot libraries and our plots were created using the PyLab Python libraries, our networks were visualised in Cytoscape 1.5 [31].
